# Incremental Value of Sestamibi SPECT/CT Over Dual-Phase Planar Scintigraphy in Patients With Primary Hyperparathyroidism and Inconclusive Ultrasound

**DOI:** 10.3389/fmed.2019.00164

**Published:** 2019-07-16

**Authors:** Roberta Assante, Emilia Zampella, Emanuele Nicolai, Wanda Acampa, Emilia Vergara, Carmela Nappi, Valeria Gaudieri, Giovanni Fiumara, Michele Klain, Mario Petretta, Alberto Cuocolo

**Affiliations:** ^1^Department of Advanced Biomedical Sciences, University Federico II, Naples, Italy; ^2^IRCCS-SDN, Naples, Italy; ^3^Department of Translational Medical Sciences, University Federico II, Naples, Italy

**Keywords:** hyperparathyroidism, ultrasound, sestamibi, planar imaging, SPECT/CT

## Abstract

**Background:** We evaluated the incremental value of [^99m^Tc]sestamibi single photon-emission computed tomography (SPECT)/computed tomography (CT) over planar imaging for localization of abnormal parathyroid tissue in patients with primary hyperparathyroidism.

**Methods:** Forty-six patients with biochemical evidence of hyperparathyroidism and inconclusive ultrasound underwent sestamibi dual-phase planar scintigraphy and SPECT/CT for preoperative localization of parathyroid adenoma. Imaging findings were compared with histopathological data. Decision tree analysis was performed to evaluate the value of SPECT/CT over planar scintigraphy for classifying patients with or without hyperfunctioning parathyroid tissue. The added value of SPECT/CT was also evaluated by decision curve analysis.

**Results:** Planar scintigraphy was positive for presence of hyperfunctioning parathyroid in 52% of patients, with sensitivity of 63% and specificity of 100%. SPECT/CT was positive in 80% of patients with sensitivity of 97% and specificity of 100%. At decision tree analysis, after an initial split on planar imaging results, no further split was performed in patients with positive results, while those with negative results were further stratified by SPECT/CT. At decision curve analysis, the model including SPECT/CT was associated with the highest net benefit compared to the model including only planar technique and to a strategy considering that all patients should be treated.

**Conclusion:** Sestamibi SPECT/CT provides incremental value over dual-phase scintigraphy in preoperative localization of hyperfunctioning parathyroid tissue in subjects with inconclusive ultrasound. Hybrid technique allows a better identification of pathological lesion to perform minimally invasive surgery and showed the highest net benefit, improving selection of surgical approach.

## Introduction

Primary hyperparathyroidism is an endocrine disorder with high prevalence characterized by an increased production of parathyroid hormone (PTH) from autonomous parathyroid tissue no longer responsive to physiological feed-back from serum calcium levels (1). Therefore, it is frequently coupled with an augmented level of total serum calcium and PTH. In some patients PTH levels may be normal but inappropriate to hypercalcemia (2, 3). Primary hyperparathyroidism is typically caused by a single parathyroid adenoma (about 90%), less frequently by hyperplasia of the glands (about 6%) or multiple adenomas (about 4%), and rarely (less of 1%) by parathyroid carcinoma (4). Conventional surgery has consisted in bilateral exploration with identification of all parathyroid glands (5). The current trend is toward minimally invasive parathyroid surgery, whenever this strategy appears appropriate. The success of minimally invasive parathyroid surgery not only depends on an experienced surgeon, but also on a sensitive and accurate imaging technique for parathyroid tissue localization (6).

The most prevalent approach for preoperative evaluation is combining ultrasound with sestamibi scintigraphy (3, 7, 8). Scintigraphy is less operator-dependent than ultrasound and is an effective way to visualize ectopic glands, whereas sonography allows simultaneous evaluation of the thyroid and fine-needle aspiration biopsy of suspicious lesions. Both are widely available and relatively inexpensive. A stepwise approach has been proposed (9). Either ultrasound or sestamibi scintigraphy is performed initially; if either result is definitive, minimally invasive parathyroidectomy may be performed. On the other hand, if both tests are inconclusive or contradictory, computed tomography (CT) should be considered. Hybrid imaging with single photon-emission computed tomography (SPECT)/CT, which combines anatomical and functional datasets has become increasingly available in the last years (10–12). Prior studies compared different imaging approaches for preoperative localization of parathyroid adenomas. In primary hyperparathyroidism the diagnostic accuracy values of sestamibi scintigraphy show a range of 65–97% that increases with association of SPECT imaging (13, 14). Integration of tomographic technique is particularly useful in presence of ectopic glandular, multiglandular suspected disease, concomitant nodular thyroid disease and recurrence or persistence of disease after surgery (15–17). Hybrid SPECT/CT approach is superior to SPECT alone for parathyroid lesion localization, especially in presence of mediastinal focus, due to the ability of the technique to better characterize SPECT findings based on their corresponding CT findings (10, 11, 18–20). Studies comparing SPECT/CT to either planar or planar/SPECT techniques report incremental diagnostic value for SPECT/CT. A recent meta-analysis demonstrated that SPECT/CT had a superior diagnostic efficacy in detecting and localizing diseased parathyroid glands when compared with SPECT and planar imaging (21). However, the clinical impact and net benefit of SPEC/CT on patient management have not been yet investigated. In addition, there is no defined consensus on the best preoperative imaging protocol for targeted, faster and less invasive intervention, and greatly reducing the risk of re-intervention.

The aim of this study was to evaluate the incremental value and the clinical implications of sestamibi SPECT/CT imaging over planar dual-phase technique for pre-surgical localization of abnormal parathyroid glands in patients with primary hyperparathyroidism and inconclusive ultrasound.

## Materials and Methods

### Patients

We conducted a retrospective review of 46 consecutive patients with biochemical evidence of primary hyperparathyroidism and inconclusive ultrasound referred to radionuclide imaging for preoperative localization of parathyroid adenoma or hyperplasia at a single institution from April 2015 to August 2017. Diagnosis of primary hyperparathyroidism was based on elevated serum calcium levels (>10.5 mg/dL), with exclusion of other secondary causes of hypercalcemia, accompanied by elevated PTH levels (>65 pg/mL). All patients underwent neck ultrasound before the parathyroid radionuclide imaging. Patients were addressed to surgery on the basis of recommendation of the American Association of Endocrine Surgeons (22). The combined imaging report was used for surgical planning, with review of the images performed at the discretion of the surgeon. Pathologic examination of resected parathyroid glands was performed to confirm parathyroid adenoma or hyperplasia. The reference standard for correct localization was the location of the gland or glands on the basis of surgical reports, with pathologic confirmation of resected glands as parathyroid adenoma or hyperplasia. The imaging data were based on retrospective review of the medical reports, and no reinterpretation of the images was performed. The review committee of our institution approved the study and all patient gave informed consent.

### Image Acquisition

All patients underwent planar and SPECT/CT imaging after intravenous injection of 740 MBq of [^99m^Tc]sestamibi according to the recommendations of the European Association of Nuclear Medicine (6). For planar imaging, digital data were acquired in a 128 × 128 matrix using a low-energy, high-resolution, parallel hole collimator. Planar images included anterior views of the neck and the upper thorax with the patient in the supine position and the neck supported in an extended position and with arms down. Early (15 min post-injection) and delayed (2 h post-injection) high count images (at least 600 s/per image) were obtained. SPECT/CT hybrid imaging was performed after the acquisition of delayed planar images. No oral or intravenous contrast material was used. Imaging procedures were performed using a 16-section SPECT/CT scanner (Symbia T; Siemens, Erlangen, Germany). The x-axis field of view was from the level of the parotid glands to the heart. Patients were positioned supine, with the neck supported in an extended position and arms lowered alongside the body. SPECT images were acquired with a 128 × 128 matrix by using a magnification of 1.003, with a 15% window centered around a 140-keV photopeak by using a low-energy, high-spatial-resolution parallel hole collimator. A step-and-shoot protocol was used and consisted of 20 s per frame with a total of 64 frames. Transverse, coronal, and sagittal SPECT images were generated by using a Gaussian 2.0 prefilter, and they were post-processed by using fast low-angle shot three-dimensional iterative reconstruction (four iterations, eight subsets). An attenuation correction factor of 0.15/cm was applied with the Chang method. The non-contrast CT portion of the study was done using 120 kV, 80 mA, and 2.5 mm slice thickness.

### Image Interpretation

Ultrasound studies were performed and interpreted by two experienced imaging specialists (EV and MK) who were not aware of the parathyroid scintigraphy findings. Ultrasound was considered inconclusive when led to no concordant definite results either negative or positive by the two observers. Planar and SPECT/CT images were visually inspected by two experienced nuclear medicine physicians (RA and EZ). The same readers evaluated both planar and SPECT/CT acquisition. For planar imaging, focal areas of increased tracer activity, which showed either a relative progressive increase over time or a fixed uptake that persisted on delayed imaging, was considered pathological hyperfunctioning parathyroid glands (6). For SPECT/CT imaging, the positive criteria included the presence of an oval formation with smooth margins or slightly lobulated, with lower density compared to the thyroid parenchyma and size more than 0.8 cm of largest diameter associated with increased tracer activity (23).

### Statistical Analysis

The diagnostic value of imaging findings was assessed by comparison with histopathological data and/or follow-up data. Sensitivity and specificity were calculated according to standard formulas (24). Concordance of diagnostic performance between planar and SPECT/CT imaging was assessed by the κ statistic. A κ value ≤0.40, between 0.41 and 0.60, between 0.61 to 0.80 and >0.80 indicated poor, moderate, good and excellent agreement, respectively. Decision tree analysis was performed to evaluate the added diagnostic value of SPECT/CT over planar imaging. In particular, a conditional inference tree was built using the ctree function of party package (R software), as described by Zhang et al. (25). The tree is made up of decision nodes, branches and leaf nodes, placed upside down, so the root is at the top and leaves indicating an outcome category is put at the bottom. At the root, all classifications are mixed, representing the original dataset. Then the tree grows to the first node where a certain feature variable is used to split the population into categories. Decision curve analysis (26) was also performed to obtain the net benefit curves using planar imaging and SPECT/CT, respectively; the statistical difference in the trapezoidal area of two net benefit curves was calculated using 1,000 bootstrap replications as proposed by Zhang et al. (27) over the probability threshold range 0.1–0.9. Statistical analysis was performed using Stata 15 (StataCorp, College Station, Texas USA) and R software (version 3.5.3) (28).

## Results

Table 1 summarizes the patient demographics. The mean age of the patient cohort was 57 ± 14 years (age range, 24–80 years). Of the 46 patients, 36 patients (78%) were women and 10 (28%) were men. Twenty-four patients had history of thyroid disease: nodular type in 18 and inflammatory type in 4; two patients presented residual thyroid tissue after total thyroidectomy. The median calcium level was 10.9 mg/dL (interquartile range, 10.5–11.2 mg/dL) and median PTH level was 91.5 pg/mL (interquartile range, 76.4–131.4); normal range for serum calcium was 8.4–10.5 mg/dL and normal range for parathyroid hormone was 10–65 pg/mL. Baseline patient characteristics, including calcium and PTH levels, were unrelated to the imaging findings and surgical outcomes.

**Table 1:** Patient demographics and baseline characteristics.

**Parameter**	**Value**
Mean age (years)^*^	57 ± 14 (24–80)
**Gender**	
Women (*n*)	36
Men (*n*)	10
**History of thyroid disease**	
Nodular type (*n*)	18
Inflammatory type	4
Residual thyroid tissue after total thyroidectomy	2
**Biochemical profile^†^**	
Median serum parathyroid hormone (pg/mL)	91.5 (76.4–131.4)
Median serum calcium (mg/dL)	10.9 (10.5–11.2)

*Normal ranges for parathyroid hormone and serum calcium are 10–65 pg/mL and 8.4–10.5 mg/dL, respectively*.

* *Data are mean ± standard deviation; data in parentheses are range*.

† *Data in parentheses are interquartile range*.

### Imaging Findings

Planar scintigraphy resulted positive for presence of hyperfunctioning parathyroid in 24 (52%) and negative in 22 (48%) patients. SPECT/CT imaging was positive in 37 (80%) and negative in 9 (20%) patients. Patients with a positive result at planar (*n* = 24) and/or SPECT/CT (*n* = 14) study underwent surgery. Of these 38 patients, 36 patients (95%) had solitary parathyroid adenoma and 2 patients (5%) had glandular hyperplasia. At day 1 postoperatively, PTH serum level decreased in all patients. The median decrease was 79% (from 14 to 99%).

**Figure 1:**
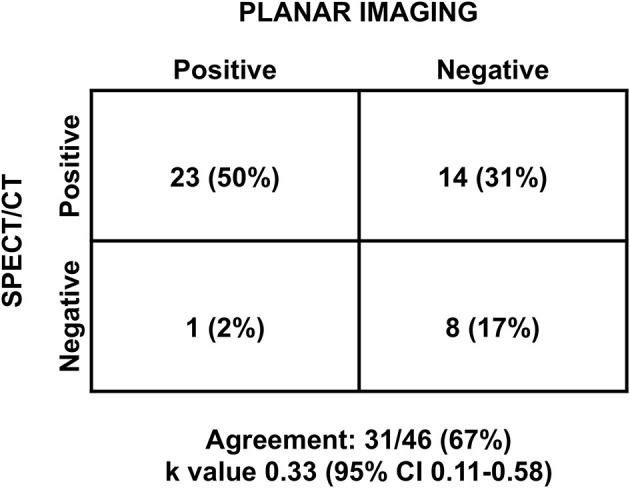
Diagnostic agreement between planar and SPECT/CT imaging, in the overall population. CI, confidence interval.

**Figure 2:**
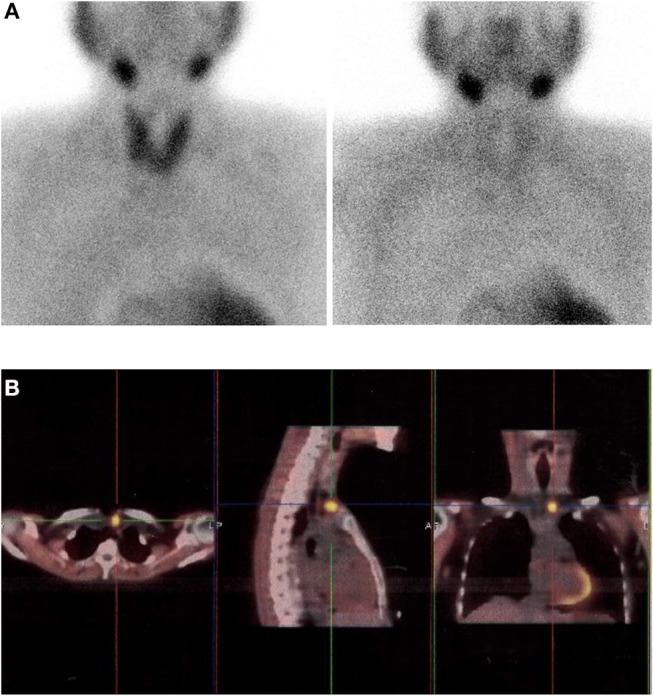
Images in a 63-year-old woman with primary hyperparathyroidism. **(A)** Dual-phase planar parathyroid images show diffuse sestamibi uptake in the thyroid gland, but no focal tracer uptake on early acquisition and uniform washout on delayed acquisition. **(B)** Axial, sagittal and coronal delayed-phase sestamibi SPECT/CT images show focal sestamibi uptake localizing to a single abnormal left upper parathyroid gland. The patient underwent minimally invasive parathyroidectomy for a single left upper parathyroid adenoma and experienced an appropriate drop in intraoperative parathyroid hormone.

SPECT/CT was able to identify hyperfunctioning parathyroid tissue in 14 patients with negative planar scintigraphy. Only one patient resulted as negative at SPECT/CT was positive at planar study and considered false negative. Thus, SPECT/CT localized abnormal parathyroid glands with 97% sensitivity and 100% specificity, whereas planar imaging showed values of 63 and 100%, respectively. Agreement between planar scintigraphy and SPECT/CT imaging for the classification of patients as positive or negative in the overall study population is depicted in Figure 1. Overall concordance of diagnostic performance between the two methods was observed in 31 (67%) patients with a κ value of 0.33. Figure 2 show example of the modalities in a patient with hyperparathyroidism with negative planar scintigraphy and positive SPECT/CT imaging.

**Figure 3:**
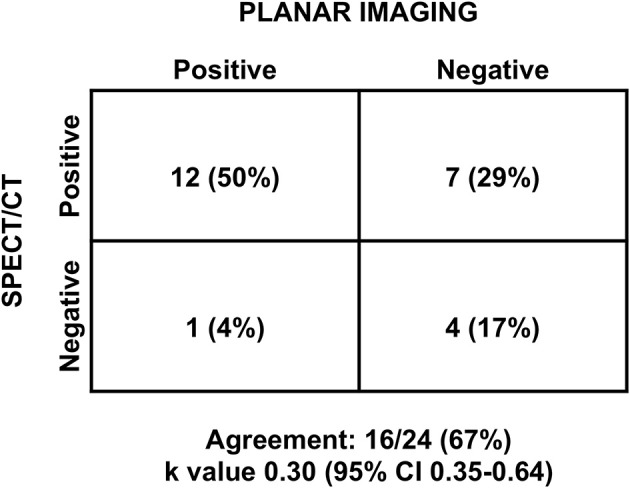
Diagnostic agreement between planar and SPECT/CT imaging, in patients with concomitant thyroid disease. CI, confidence interval.

Considering the 24 patients with concomitant thyroid disease, planar scintigraphy showed abnormal parathyroid tissue in 13 (54%) patients. Conversely, SPECT/CT showed a positive result for pathological hyperfunctioning parathyroid in 19 (79%) patients. In this subgroup of patients, concordance of diagnostic performance between planar and SPECT/CT imaging was observed in 16 (67%) patients with a κ value of 0.30 (Figure 3).

Planar scintigraphy and SPECT/CT imaging resulted negative for presence of hyperfunctioning parathyroid in 8 patients (17%). After a 6-month follow-up, PTH serum level of these patients normalized; serum PTH level decreased from 87.6 ± 43.2 to 46.3 ± 7.3 pg/mL (*p* < 0.01), and serum calcium level changed from 8.5 ± 0.4 to 8.8 ± 0.3 mg/dL (*p* = 0.1). The final clinical diagnosis for these patients was secondary hyperparathyroidism.

### Decision Tree Analysis

**Figure 4:**
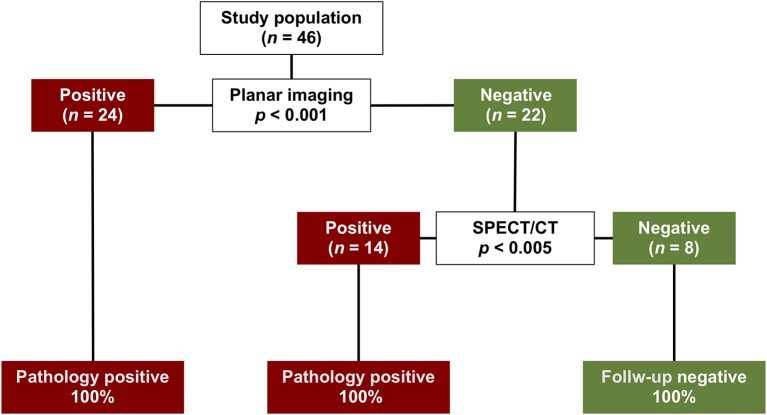
Decision tree analysis for the classification of patients as positive or negative based on planar and SPECT/CT results. The split produced three terminal groups. The initial split was on planar imaging results. In patients with positive results no further split was performed, while those with negative results were further stratified by SPECT/CT. For each split, the *p* value testing the null hypothesis of independence between the input variables and the response is depicted.

**Figure 5:**
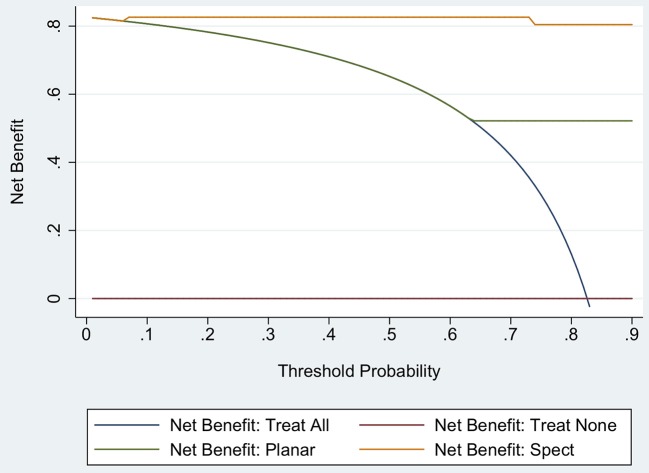
Decision curves analysis using a model including planar imaging and a model including SPECT/CT. The red line is the net benefit of treating no patients, assuming that none had hyperfunctioning parathyroid tissue; the navy line is the net benefit of treating all patients assuming that all had hyperfunctioning parathyroid tissue; the green line is the net benefit of treating patients according to the model including planar imaging; and the orange line is the net benefit of treating patients based on the model with SPECT/CT.

Results of decision tree analysis based on planar and SPECT/CT imaging are depicted in Figure 4. As shown, the analysis produced three terminal groups. In particular, after an initial split based on planar imaging results, in patients with positive results no further split was performed, while those with negative results were further stratified by SPECT/CT. At decision curve analysis, model with SPECT/CT was associated with the highest net benefit over a range of threshold probabilities up to 90% compared to the model including only planar technique and to a strategy considering that all patients need to be treated (Figure 5). The difference in the net benefit curve areas of the SPECT/CT model and the planar technique was statistically significant, with a global *p*-value of 0.02 in the threshold range from 0.1 to 0.9.

## Discussion

From this study it emerged that SPECT/CT provide incremental diagnostic value over dual phase scintigraphy for preoperative localization of hyperfunctioning parathyroid tissue in patients with primary hyperparathyroidism and inconclusive ultrasound. Indeed, hybrid imaging provided additional data in 30% of patients. SPECT/CT allow accurate localization to perform minimally invasive surgery. Surgery represents the only definitive treatment recommended of the primary hyperparathyroidism and the elective approach in most patients with this disease. The classic bilateral exploration of the neck used to identify all parathyroid glands has been replaced by image-guided surgery (29).

The availability of several imaging methods that allow preoperative localization of abnormal parathyroid tissue has led to the frequent use of an increasingly invasive surgical approach and mini-invasive surgical procedures are becoming more and more precise. For successful minimally invasive parathyroid surgery, accurate preoperative localization is necessary.

The development of SPECT/CT hybrid devices, consisting of a traditional camera integrated with a CT scanner in a single device, allowed the integration of SPECT's high sensitivity and precision by using scintigraphic tomography images and the precise anatomical definition of CT images. This combination of anatomical and functional information led to a high sensitivity and specificity. Parathyroid scintigraphy with sestamibi using the “dual phase” technique represents established reliable and cost-effective imaging method for diagnosing parathyroid adenoma or hyperplasia (11). However, scintigraphic scanning with planar acquisitions has lower sensitivity. From the data reported in the literature, the sensitivity of planar images, SPECT and SPECT/CT in the preoperative evaluation in patients was 63, 66, 84%, respectively, and the positive predictive value was 90, 82, and 95%, respectively (13). In our population, SPECT/CT had a sensitivity of 97%, compared with 63% for planar imaging. A recent meta-analysis demonstrated that sestamibi SPECT/CT is a very accurate method for detecting hyperfunctioning parathyroid glands in patients with primary hyperparathyroidism, with overall detection rate of 88% both on a per-patient-based and a per-lesion-based analysis (30). In the present study, the agreement between planar and SPECT/CT imaging in classifying patients as positive or negative was poor with a κ value <0.40, both in the overall population and in patients with concomitant thyroid disease.

Use of SPECT/CT allowed better identification of parathyroid lesions and showed the highest net benefit, improving selection and clinical impact of surgical approach. Decision tree analysis also indicates that the addition of SPECT/CT is useful among patients with previous negative planar results, to better discriminate between positive and negative patients, leading to a more accurate preoperative diagnosis. Conversely, in subject with positive results at planar study, other diagnostic procedures are not recommended. Interestingly, use of SPECT/CT led to a significant (*p* = 0.02) increase in the area under the decision-curve analysis as compared to planar imaging.

In the present study, SPECT/CT allowed to detect hyperfunctioning parathyroid tissue in 14 (64%) of the 22 patients with negative planar scintigraphy, allowing appropriate surgical intervention. The integration between functional and morphological data with hybrid devices may also allow greater diagnostic accuracy in conditions where planar technique is less accurate, such as patients with a concomitant thyroid pathology (18, 31). Dual-radiopharmaceutical subtraction imaging can be performed in conjunction with dual-phase planar parathyroid imaging (6). This technique involves planar imaging of the thyroid gland defined by [^123^I]sodium iodide or [^99m^Tc]pertechnetate, and subsequently subtracting out the thyroid activity from the combined [^99m^Tc]sestamibi thyroid and parathyroid images. The subtraction strategy might provide additional information in patients with concomitant thyroid disease. However, the Society of Nuclear Medicine and Molecular Imaging (32) guidelines on parathyroid scintigraphy discussed the dual-phase and subtraction approach and stated that no diagnostic superiority was observed among them. In our study, among the 24 patients with concomitant thyroid disease, SPECT/CT allowed to detect hyperfunctioning parathyroid tissue in 6 (54%) of the 11 patients with negative planar scintigraphy.

In patients with ectopic parathyroid adenoma in the mediastinum or with anatomic neck anatomies, SPECT/TC may provide more information, giving the exact anatomical location of the anomaly (10). Hybrid imaging through CT improves not only photon attenuation correction, but also allows for easier correlation of districts with physiological variants or abnormal tracer accumulation based on anatomic reference points improving functional and metabolic information provided by the scintigraphic image (33, 34). Indeed, in presence of ectasia glandular, SPECT allows both the identification of pathologic tissue and the determination of the depth of the hyperactive parathyroid (16). One patient among those we studied presented an accumulation of the mediastinum tracer in SPECT/CT, which the planar technique did not detect. Magnetic resonance imaging performed for integration confirmed the report of the tomographic method and then patient underwent to a resolving surgical procedure. According to current guidelines (6), CT and magnetic resonance a represent II level examinations and are predominantly indicated in patients already undergoing parathyroidectomy that show relapse or persistence of the disease. In these patients, the ability to integrate the scintigraphic tomographic data with the anatomical detail of CT allows for a single examination to overcome the diagnostic limits of the only functional exam with economical and time-saving benefits for patient (35, 36).

Our study had some limitations, including the retrospective design and the small number of patients that may have an impact on the presence of poor concordance between planar and SPECT/CT imaging results. In addition, the lack of operative results for patients with negative imaging limits the complete evaluation of false negative findings. However, considering the increasing trust of surgeons in parathyroid nuclear scans and the adoption of minimally invasive surgery, it would be difficult to conduct a prospective study in which patients with negative scan results undergo surgery. Finally, all SPECT/CT studies were acquired without use of contrast enhancement.

## Conclusions

Sestamibi SPECT/CT provides incremental value over dual phase scintigraphy in preoperative evaluation of hyperfunctioning parathyroid tissue in patients with primary hyperparathyroidism and inconclusive ultrasound. Use of hybrid technique allows a better identification of pathological lesion to perform minimally invasive surgery showed the highest net benefit, improving selection and clinical impact of surgical approach.

## Data Availability

The raw data supporting the conclusions of this manuscript will be made available by the authors, without undue reservation, to any qualified researcher.

## Ethics Statement

This study follows the principles expressed in the Declaration of Helsinki. All study participants waived informed written consents owing to the retrospective analysis, and the study design was approved by the Ethics Committee of the University of Naples Federico II.

## Author Contributions

RA, EZ, EN, WA, MP, and AC contributed conception and design of the study. EZ, EV, EN, CN, VG, GF, and MK collected the data and organized the database. EV, CN, VG, GF, MK, and MP analyzed the data. RA wrote the first draft of the manuscript. All authors contributed to the final version of the manuscript.

### Conflict of Interest Statement

The authors declare that the research was conducted in the absence of any commercial or financial relationships that could be construed as a potential conflict of interest.
